# Centrosomal protein CP110 controls maturation of the mother centriole during cilia biogenesis

**DOI:** 10.1242/dev.130120

**Published:** 2016-05-01

**Authors:** Sharda Prasad Yadav, Neel Kamal Sharma, Chunqiao Liu, Lijin Dong, Tiansen Li, Anand Swaroop

**Affiliations:** 1 Neurobiology-Neurodegeneration and Repair Laboratory, National Eye Institute, National Institutes of Health, Bethesda, MD 20892, USA; 2 Genetic Engineering Core, National Eye Institute, National Institutes of Health, Bethesda, MD 20892, USA

**Keywords:** Centrosome, Ciliopathies, Microtubule, Primary cilia, Organogenesis, Membrane anchoring, Ccp110, Mouse

## Abstract

Defects in cilia centrosomal genes cause pleiotropic clinical phenotypes, collectively called ciliopathies. Cilia biogenesis is initiated by the interaction of positive and negative regulators. Centriolar coiled coil protein 110 (CP110) caps the distal end of the mother centriole and is known to act as a suppressor to control the timing of ciliogenesis. Here, we demonstrate that CP110 promotes cilia formation *in vivo*, in contrast to findings in cultured cells. *Cp110^−/−^* mice die shortly after birth owing to organogenesis defects as in ciliopathies. Shh signaling is impaired in null embryos and primary cilia are reduced in multiple tissues. We show that CP110 is required for anchoring of basal bodies to the membrane during cilia formation. CP110 loss resulted in an abnormal distribution of core components of subdistal appendages (SDAs) and of recycling endosomes, which may be associated with premature extension of axonemal microtubules. Our data implicate CP110 in SDA assembly and ciliary vesicle docking, two requisite early steps in cilia formation. We suggest that CP110 has unique context-dependent functions, acting as both a suppressor and a promoter of ciliogenesis.

## Abstract (precis)

**Highlighted article:** CP110 promotes the assembly of subdistal appendages and ciliary vesicle docking during cilia formation *in vivo*, thereby facilitating mammalian organogenesis.

## INTRODUCTION

The primary cilium is a near ubiquitous microtubule (MT)-based organelle that protrudes from the cell surface of most vertebrate cells. This antenna-like organelle segregates signal transduction machineries that are crucial for organogenesis and tissue homeostasis (Eggenschwiler and Anderson, 2007; Gerdes et al., 2009; Singla and Reiter, 2006). Impaired cilia biogenesis and/or function impact the development of multiple organs resulting in pleiotropic phenotypes, collectively termed ciliopathies (Hildebrandt et al., 2011; Nigg and Raff, 2009; Novarino et al., 2011). These phenotypes can include retinal degeneration, cognitive impairment, neural tube defects, hydrocephalus, polycystic kidneys, polydactyly, situs inversus and obesity. Depending on the combination of defects, ciliopathies can be categorized clinically as distinct disorders, such as Bardet–Biedl syndrome (BBS), Joubert syndrome (JBTS), Senior-Loken syndrome, Jeune asphyxiating thoracic dystrophy, or a rare lethal malformation called Meckel syndrome (MKS). Strikingly, a specific syndromic disorder can result from mutations in different genes, and mutations in one gene can lead to distinct clinical manifestations. The observed clinical diversity in ciliopathies might reflect a cumulative genetic load of variants/mutations and interactions among cilia centrosome-associated genes (Novarino et al., 2011; Sang et al., 2011; Zaghloul and Katsanis, 2010).

Ciliogenesis occurs as cells exit from the cell cycle, whereupon centrioles migrate to the apical domain and become basal bodies, which subsequently serve to anchor the nascent cilium. The two centrioles in the centrosome are structurally distinct; the older mother centriole (MC) is decorated with distal (DA) and subdistal (SDA) appendages (Paintrand et al., 1992) that are essential for subsequent membrane docking and anchoring to the cytoplasmic MTs (Bornens, 2002). A critical initial step in ciliogenesis is the docking of a primary ciliary vesicle to the distal end of the MC (Ishikawa and Marshall, 2011). This primary vesicle grows in size as smaller secondary vesicles are added, while the axonemal MTs lengthen and become ensheathed within the ciliary membrane. Fusion of ciliary vesicle to the cell membrane leads to the emergence of cilia from the cell surface. Recycling endosome components Rab11, Rab11 GTPase-activating protein Evi5, and the exocyst components Sec15 (Exoc6) and Sec6 (Exoc3) participate in the growth of the nascent cilia (Hehnly et al., 2012). Two SDA proteins, centriolin and Odf2 (cenexin), regulate endosomal components at the basal body (Carvalho-Santos et al., 2010; Hehnly et al., 2013). Growth of the cilium requires the orchestrated trafficking of lipids and proteins via molecular motors and intraflagellar transport (IFT) complexes (Haimo and Rosenbaum, 1981; Pazour et al., 2005). Once transported to the ciliary base, the cargo must cross the transition zone, a region between the basal body and the axoneme. A transition zone complex containing Tctn1, Tctn3 and seven MKS proteins (Mks1, Tmem216, Tmem67, Cep290, Cc2d2a, B9d1 and Tctn2) has been demonstrated to regulate ciliogenesis and ciliary membrane composition in a tissue-dependent manner (Garcia-Gonzalo et al., 2011). Almost 300 proteins can associate with the basal body and the cilium at different stages of ciliogenesis and sensory signaling (Gherman et al., 2006; Pazour et al., 2005).

MC-associated positive and negative regulators are essential for cilia formation (Hergovich et al., 2009; Joo et al., 2013; Sharma et al., 2012). A key suppressor of ciliogenesis is centriolar coiled coil protein 110 (CP110; also known as Ccp110), which was isolated in a screen to identify substrates for cyclin-dependent kinases and shown to have a role in regulating centrosome duplication and separation (Chen et al., 2002). CP110 is strongly induced as cells enter S phase and downregulated at G2/M and G1 phase. CP110 caps the distal ends of both centrioles in non-ciliated cells. As cells begin to develop a cilium, CP110 is removed from the MC and degraded through ubiquitylation but remains at the distal end of the daughter centriole. The removal of CP110 coincides with cilia formation (Chen et al., 2002; Schmidt et al., 2009) and, along with recruitment of Ttbk2 to the distal MC, is one of the earliest identifiable events in ciliogenesis (Goetz et al., 2012). Knockdown of CP110 leads to the premature formation of aberrant cilia, and its overexpression appears to suppress ciliogenesis. As such, CP110 is recognized as a crucial, negative regulator of cilia biogenesis.

Although *in vitro* studies have linked CP110 to the suppression of cilia biogenesis, the molecular mechanism of how CP110 executes its suppressive effect is not clearly elucidated. CP110 could suppress ciliogenesis by acting simply as a passive physical barrier and by capping the growing ends of MTs to prevent their elongation. Alternatively, CP110 might have a more complex role in regulating ciliogenesis. For example, CP110 binds to and antagonizes the function of Cep290, a positive regulator of ciliogenesis (Tsang et al., 2008). However, ablation of *Cep290* in mouse cells did not abolish ciliogenesis and, instead, longer, functionally defective cilia were produced (Rachel et al., 2015); therefore, antagonizing Cep290 cannot be the basis of ciliogenesis suppression by CP110. If CP110 acts primarily as a barrier to axonemal MT elongation, its removal alone should be sufficient for spontaneous cilia formation. Although depletion of CP110 alone seemed sufficient to promote ectopic cilia formation in some studies (Spektor et al., 2007; Tsang et al., 2009), others reported abnormal elongation of centriolar MTs (Franz et al., 2013; Schmidt et al., 2009) that were not encapsulated within a membrane and lacked other features of primary cilia (Schmidt et al., 2009; Tang et al., 2009). Importantly, such aberrant centrioles were not docked to ciliary vesicles, a requisite step in early ciliogenesis. Complete removal of CP110 in *Drosophila* causes centriolar MT elongation but does not induce ectopic cilia formation (Franz et al., 2013). Taken together, it appears that loss of CP110 by itself is insufficient to initiate cilia biogenesis and that other crucial suppressors may keep cilia formation in check in the absence of CP110. Notably, CP110 is involved in a complex protein interaction network, and some of the interacting proteins, such as Cep97 and Kif24, are indeed suppressors of cilia formation (Kobayashi et al., 2011; Spektor et al., 2007). Interestingly, CP110 also strongly interacts with proteins, including Cep290, Cep104 and Talpid3 (2700049A03Rik) (Jiang et al., 2012; Tsang et al., 2008), that are key positive regulators of ciliogenesis.

To gain a more complete understanding of its function, we generated mice with complete loss of CP110. Unexpectedly, *Cp110^−/−^* mice manifest pleiotropic phenotypes that can collectively be attributed to failure of cilia formation. Our study thus uncovers previously unappreciated aspects of CP110 function in primary cilia biogenesis.

## RESULTS

### Organogenesis defects in mice with complete loss of CP110

**Fig. 1.**
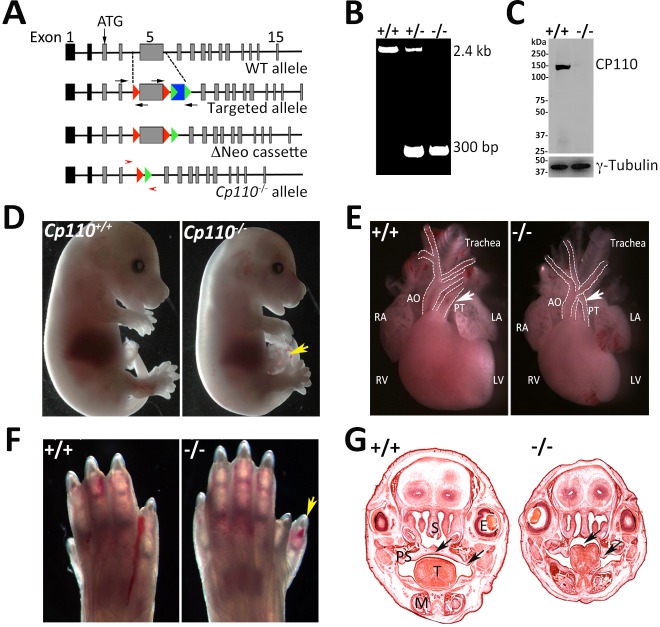
**Loss of CP110 leads to developmental defects reminiscent of ciliopathy mutations.** (A) Generation of *Cp110^−/−^* mice. Schematic showing the wild-type (WT) mouse *Cp110* gene, the targeted allele with the neomycin resistance cassette (blue) flanked by FRT fragments (green arrowheads), the neomycin cassette removed (ΔNeo), and with exon 5 of *Cp110* deleted; loxP fragments (large red arrowheads) were located on either side of exon 5 to delete it from the targeted allele (exon information from ENSEMBL: ENSMUST00000106557). Black arrows indicate the primers used for long-range PCR to confirm the integration of the cassette in ESCs and in the founder mouse line. (B) Genotype analysis of wild-type, *Cp110* heterozygous and null pups using forward and reverse primers (small red arrowheads) located on either side of exon 5 as indicated in A. In wild-type animals a 2.4 kb fragment was detected, whereas *Cp110^−/−^* animals showed only a 300 bp product after exon 5 deletion. (C) Immunoblot analysis with anti-CP110 antibody on protein extracts from E14.5 wild-type and *Cp110^−/−^* embryos. A specific band for CP110 was detected between 110 and 150 kDa because of its post-translational modifications. γ-tubulin was used as a loading control. (D) Gross phenotype of E14.5 wild-type and *Cp110^−/−^* embryos. The mutants were smaller and showed omphalocoel (arrow). (E) P0 heart of wild-type and *Cp110^−/−^* pups, showing truncus arteriosus in the mutant (compare at arrows). White dotted lines trace the major arteries. RA, right auricle; LA, left auricle; RV, right ventricle; LV, left ventricle; AO, aorta; PT, pulmonary trunk. (F) P0 limb of wild-type and *Cp110^−/−^* pups. We observed polydactyly (arrow) in almost 30% of *Cp110^−/−^* pups. (G) Hematoxylin and Eosin (H&E) staining of coronal sections of the head of wild-type and *Cp110^−/−^* at E16.5. Arrows indicate cleft palate in *Cp110^−/−^* animals. T, tongue; E, eye; M, mandible; PS, para-sphenoid; S, sphenoid.

The mouse *Cp110* (*Ccp110*) gene contains 16 exons and encodes five different transcripts (based on the UCSC mouse genome browser), the longest of which is translated into a protein of 1004 amino acid residues. To eliminate the protein-coding transcript(s), we engineered a targeting vector containing exon 5 flanked with flox sites and with a neomycin selection cassette flanked with FRT sites (Fig. 1A). We carried out standard homologous recombination in mouse embryonic stem cells (ESCs) and selected positive clones after neomycin treatment and validation by long-range PCR. Two positive ESC clones were used for blastocyst injections to generate knockout mice. The progeny were backcrossed for five generations to the C57BL/6J mouse line to dilute any unwanted gene alleles from the ESCs. The neomycin resistance cassette was removed by breeding heterozygous floxed animals with actin-Flp recombinase mice. The neomycin-deleted animals were crossed with Zp3-Cre mice to completely ablate *Cp110* exon 5 during oogenesis (Lewandoski et al., 1997). The *Cp110^+/−^* mutants, thus obtained, were viable and bred normally. Sibling mating among *Cp110^+/−^* mice produced homozygous knockout animals, as indicated by PCR (Fig. 1B). We confirmed that homozygous pups had no CP110 protein by immunoblotting using anti-CP110 antibody (Fig. 1C).

The *Cp110^−/−^* pups were born at the expected Mendelian ratio but died within a few hours of birth. Detailed analysis of mutant pups at postnatal day (P) 0 revealed a multitude of developmental defects (Fig. 1D-G, Fig. S1A-E). *Cp110^−/−^* pups were smaller than heterozygous and wild-type littermates. Approximately 30% of *Cp110^−/−^* pups showed polydactyly and omphalocoele, an abdominal wall defect that leaves internal organs exposed. We observed severe malformation of the heart that included inversion of major blood vessels (truncus arteriosus) and ventricular septum defect, which would lead to mixing of oxygenated and deoxygenated blood and was the likely cause of perinatal death. *Cp110^−/−^* animals did not develop palatal shelves and showed fusion of medial edge epithelia leading to a cleft palate at embryonic day (E) 16.5 (Fig. 1G).

**Fig. 2.**
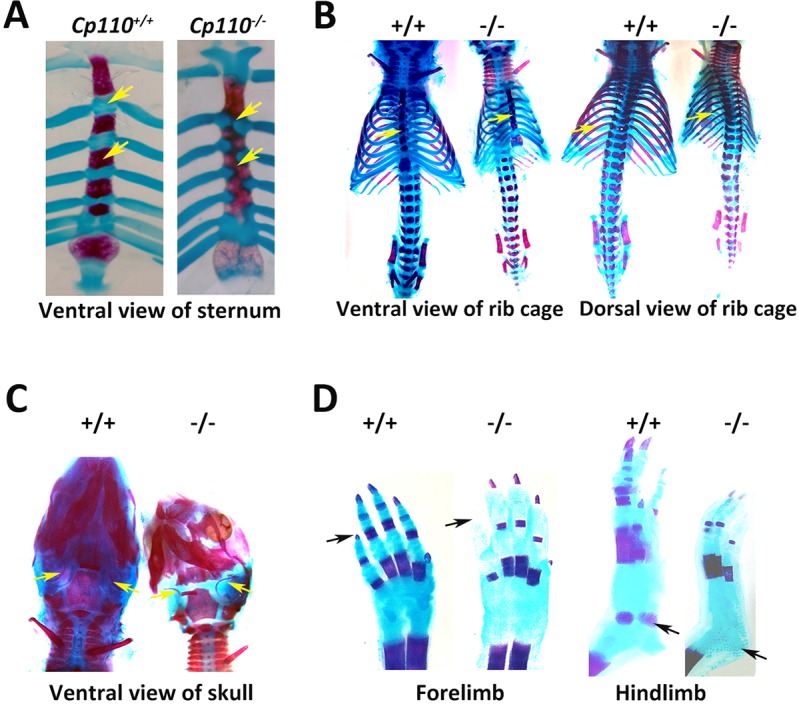
**Loss of CP110 leads to major skeletal defects.** *Cp110^−/−^* animals showed major skeletal defects and had a short rib cage, which is characteristic of Jeune syndrome. (A) P0 sternum and ribs of wild type (left) and *Cp110^−/−^* (right) showing cartilage (Alcian Blue) and bone (Alizarin Red) staining. Fusion of all four sternebrae was detected only in *Cp110^−/−^* pups (compare at arrows). This phenotype was highly penetrant, ranging from fusion of all four sternebrae to only two. (B) Alcian Blue and Alizarin Red staining of P0 pups showing the thoracic region and the rest of the lower region in ventral and dorsal view. Note the fusion of the sternebrae (compare at arrows) and shorter rib cage in the mutant. (C) Gross appearance of P0 wild-type (left) and *Cp110^−/−^* (right) skull in ventral view. Arrows mark the tympanic bone, which shows less mineralization in the mutant compared with the wild type. (D) Alcian Blue and Alizarin Red staining of P0 wild-type and *Cp110^−/−^* forelimb and hindlimb. The phalangeal and metatarsal bones (arrows) showed less mineralization in *Cp110^−/−^* animals compared with the wild type.

We observed pronounced skeletal defects in the *Cp110^−/−^* mutants. Alcian Blue (cartilage) and Alizarin Red (bone) staining of the skeletal preparations showed an increase in cartilage at the expense of bone in the *Cp110^−/−^* pups (Fig. 2). Skeletal staining revealed preaxial polydactyly in hind limbs, reduced calcification of the forelimb phalangeal bones, defect in fusion of sternebrae and cleft palate. The horizontal ribs in knockout pups were extremely short, giving rise to a constricted thoracic cavity (Fig. 2B), which might have additionally contributed to perinatal lethality due to respiratory insufficiencies. Skeletal phenotypes in the mutant were fully penetrant. We noted that the gross phenotype of mutant pups was similar to manifestations of short rib-polydactyly syndrome, a skeletal form of ciliopathy in humans caused by mutations in diverse cilia-related genes (Huber and Cormier-Daire, 2012).

### Impairment of sonic hedgehog signaling in *Cp110^−/−^* embryos

**Fig. 3.**
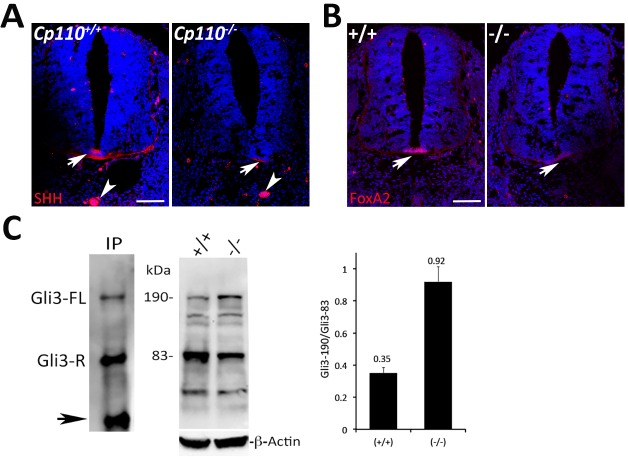
**Shh signaling is perturbed in *Cp110^−/−^* embryos.** (A,B) Immunostaining of wild-type and *Cp110^−/−^* neural tube at E11.5 for Shh (A) and the downstream transcription factor FoxA2 (B). In wild-type embryos, the Shh signal was seen in the ventral floor plate (arrow) and notochord (arrowhead), but was absent in the floor plate of *Cp110^−/−^* embryos. FoxA2 in the ventral floor plate (arrow) was reduced in the *Cp110^−/−^* embryo. Scale bars: 25 µm. (C) Immunoblot analysis with anti-Gli3 of immunoprecipitated Gli3 from *Cp110^+/−^* E10.5 embryo extract (left), showing full-length (190 kDa) and processed (83 kDa) Gli3 bands. Arrow indicates IgG heavy chain. Similar analysis was performed with wild-type and *Cp110^−/−^* E10.5 embryo extracts (right); three independent experiments were performed using embryos from three different females, and a representative immunoblot and quantification of the ratio of full-length to processed Gli3 are shown. β-actin was used as a loading control.

Sonic hedgehog (Shh) signaling plays an important role in embryonic development, and initial steps of the signaling process take place in primary cilia (Eggenschwiler and Anderson, 2007; Huangfu et al., 2003). The *Cp110^−/−^* embryos exhibited characteristic defects, including preaxial polydactyly, cardiac abnormalities and severe skeletal defects (Fig. 1D-G), reminiscent of cardinal features of ciliopathies and a perturbation in Shh signaling. We therefore examined dorsoventral patterning of the embryonic neural tube, which serves as a sensitive readout of Shh signaling (Huangfu and Anderson, 2005; Huangfu et al., 2003). Although Shh staining was comparable in the notochord between the mutant and controls at E11.5, it was significantly reduced in the floor plate of the mutant neural tube (Fig. 3A). Reduction in Shh expression correlated with a reduction in FoxA2 expression in the floor plate (Fig. 3B). These changes were observed at both the rostral and caudal levels of the neural tube. The staining pattern thus demonstrated an overall reduction in Shh signaling, as indicated by the loss of the ventralmost cell fate. Other neural tube patterning markers [e.g. HB9 (Mnx1), Nkx2.2, Islet1 (Isl1), Olig2, Pax6 and Pax7] did not reveal marked changes in expression (data not shown). The changes in the dorsoventral patterning of the neural tube were relatively mild compared with other ciliopathies but did appear similar to a number of mutants in which ciliogenesis is perturbed (Weatherbee et al., 2009; Zeng et al., 2010a).

The transcription factor Gli3 is a downstream effector of Shh signaling and can be detected in full-length (activator, 190 kDa) and processed (repressor, 83 kDa) forms (Wang et al., 2000; Zeng et al., 2010b). Gli3 processing is initiated in the cilia, hence providing an important readout for normal or defective cilia function (Eggenschwiler and Anderson, 2007). Quantification of the ratio of full-length to processed Gli3 by immunoblot analysis showed an increase in *Cp110^−/−^* compared with wild-type embryos (Fig. 3C). These results further demonstrate a defect in cilia function in mutant embryos.

### Compromised cilia formation in *Cp110^−/−^* tissues and in derivative cells

**Fig. 4.**
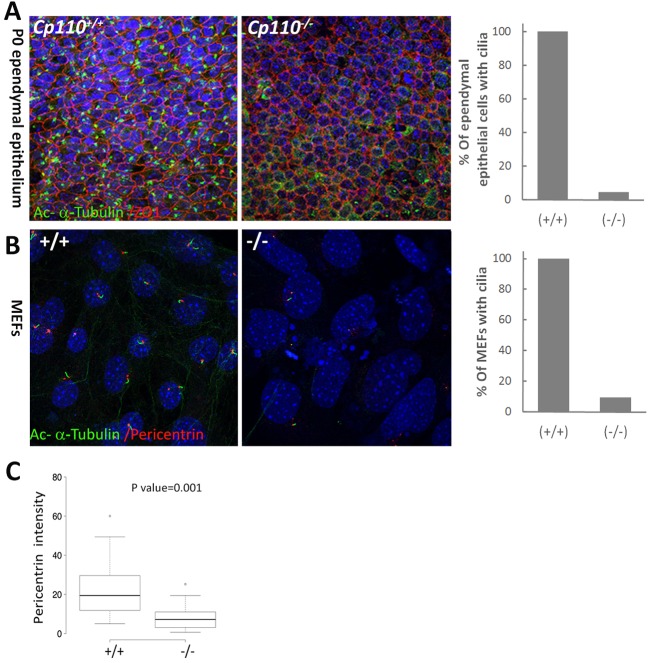
**Loss of CP110 reduces cilia formation.** (A) Immunohistochemical analysis of the ependymal epithelium of brain ventricles of P0 wild-type and *Cp110^−/−^* pups stained for acetylated α-tubulin (green) to mark cilia (*en face* view). ZO1 (red) stains the tight junctions and marks the cell boundary. Blue, DAPI. Bar chart shows the percentage of ependymal epithelial cells with cilia in the wild-type and *Cp110^−/−^* brain. (B). *Cp110^−/−^* fibroblasts failed to develop cilia. Wild-type and *Cp110^−/−^* MEFs were stained with anti-acetylated α-tubulin (green) and anti-pericentrin (red) to visualize cilia and basal bodies, respectively. Bar chart shows quantification of the percentage of MEFs with cilia. Note that axonemal staining of acetylated α-tubulin was absent from 90% of *Cp110^−/−^* MEFs. (C) Box plot showing pericentrin intensity in wild-type and *Cp110^−/−^* MEFs. The two small circles are outlier samples. Scale bars: 25 µm in A; 5 µm in B.

Given the data suggesting abnormal ciliary function in the *Cp110* mutant, we examined cilia formation directly in the mutant mouse tissues. In *en face* views of newborn *Cp110^−/−^* brains, immunostaining for acetylated α-tubulin and the cell junction protein ZO1 (Tjp1) revealed a greatly reduced number of primary cilia (less than 10% of mutant cells were ciliated) in the epithelial lining of lateral ventricles (Fig. 4A). Furthermore, we observed disruption of the unique pinwheel architecture of the ventricular surface in neurogenic regions of P0 *Cp110^−/−^* brains, suggesting aberrant neurogenesis due to reduced cilia number. Consistent with this, there was an increase in the number of mitotic nuclei in the *Cp110^−/−^* brain ventricles as well as in the neural tube (Fig. S2A), indicating aberrant cell division of the neural progenitors. Quantification of the mitotic cells in ependymal epithelia revealed the presence of ∼3% metaphase nuclei in the *Cp110^−/−^* mutant (Fig. S2B), whereas no ependymal epithelial cells were in mitosis in the wild type. Among the cells in mitosis in *Cp110^−/−^* ependymal epithelia, ∼10% had bipolar spindles and 45% had aberrant multipolar spindles (Fig. S2C), indicating supernumerary centrosomes and aneuploidy. Cilia number was reduced in *Cp110^−/−^* kidney collecting duct and embryonic neural tube compared with wild type, as indicated by staining for acetylated α-tubulin (Fig. S3A,B).

To further elucidate the role of CP110 in cilia biogenesis, we isolated mouse embryonic fibroblasts (MEFs) from null mutant embryos and tested the integrity of the basal body and axoneme structure by immunofluorescence and transmission electron microscopy (TEM). Unlike MEFs from wild-type littermates (Fig. 4B, left), *Cp110^−/−^* MEFs did not grow cilia upon serum starvation (Fig. 4B, right), as shown by the absence of acetylated α-tubulin staining, which labels the ciliary axoneme. Only ∼10% of *Cp110^−/−^* MEFs escaped this phenotype and developed cilia (Fig. 4B). There was also a significant reduction in staining for pericentrin, which marks the pericentriolar material, in *Cp110^−/−^* MEFs (Fig. 4C). Consistent with the absence of cilia, we did not detect Arl13b or centrin 1 staining in *Cp110^−/−^* MEFs (Fig. S4A). There was also a reduction in ninein staining in *Cp110^−/−^* MEFs (Fig. S4A) and increased supernumerary centrioles in a subset of mutant MEFs (Fig. S4B). These results suggested that centrioles/basal bodies are not entirely normal in the absence of CP110 and, mechanistically, an abnormal MC/basal body can lead to impaired cilia biogenesis.

### Aberrant localization of SDA and transition zone components in the basal body of *Cp110^−/−^* MEFs

**Fig. 5.**
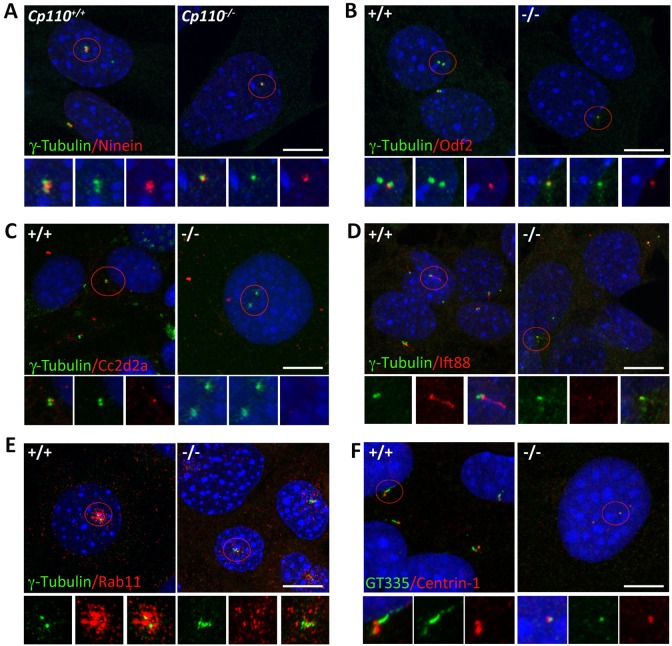
**Mislocalization of basal body-associated proteins in *Cp110^−/−^* MEFs.** (A) Staining of the MT anchoring protein ninein. Basal bodies are decorated with subdistal appendages (SDAs) that contain ninein, which helps in anchoring the MTs during cilia biogenesis. γ-tubulin (green) marks basal bodies, and SDAs are labeled with anti-ninein (red), which also stains the proximal end of both centrioles. The strong ninein staining detectable in wild-type basal bodies was reduced in *Cp110^−/−^* MEFs. Blue, DAPI. (B) Staining of the SDA assembly component Odf2, which is responsible for docking of recycling endosomes to basal bodies. Basal bodies are marked with γ-tubulin (green) and stained for Odf2 (red). We noted reduced levels and mislocalization of Odf2 staining in the mutant. (C) Staining of Cc2d2a, another SDA component. Anti-Cc2d2a labeling (red) was reduced in *Cp110^−/−^* MEFs. Basal bodies are marked with γ-tubulin (green). (D) Staining of cilia for Ift88 (red) and γ-tubulin (green) in MEFs. Ift88 staining was either absent or reduced in *Cp110^−/−^* MEFs. (E) Distribution of recycling endosomes in *Cp110^−/−^* MEFs. The basal bodies were stained for γ-tubulin (green) and recycling endosomes were labeled with anti-Rab11 (red). Punctate staining of Rab11 was observed close to basal bodies in wild-type MEFs but was reduced and dispersed in *Cp110^−/−^* MEFs. (F) Staining of MEFs for centrin 1 (red), which labels both centrioles, and polyglutamylated α-tubulin (GT335, green). GT335 was reduced in *Cp110^−/−^* MEFs. Scale bars: 25 µm.

Presence of MC but lack of ciliary axoneme suggested that CP110 is indispensable for the early ciliogenic events. After polarity-guided centriolar migration, the MC docks to the plasma membrane with DAs, whereas the anchoring of MT arrays requires SDAs. Even though MT nucleation starts with aster formation at the pericentriolar material, only the MC is able to sustain a stable MT array; this process requires ninein. Immunolabeling with anti-ninein antibody revealed a significant reduction in ninein signal at the MC in *Cp110^−/−^* MEFs (Fig. 5A, Fig. S4Ac, Fig. S5A,B). These results also indicated that proteins recruited to the SDA might be mistargeted in the absence of CP110. We also examined other known SDA components, Odf2 and Cc2d2a (mutant cells lacking these proteins do not develop cilia), by immunostaining wild-type and *Cp110^−/−^* MEFs. These proteins were reduced in distribution and intensity in the SDA of mutant MCs (Fig. 5B,C, Fig. S5C) compared with wild type, suggesting that SDAs are compromised in *Cp110^−/−^* MEFs.

We then assessed the integrity of the transition zone, which is a specialized compartment at the base of the cilium adjacent to the centriole distal end, where axonemal MTs are cross-linked to the surrounding membrane to form a barrier that gates the ciliary compartment. In general, DAs extend radial MTs, which are also known as transition fibers, that help in anchoring the basal body to the ciliary membrane and allow normal axoneme development. We stained wild-type and *Cp110^−/−^* MEFs for the transition zone marker Rpgr and the SDA protein ninein, which also labels the proximal end of the basal body and daughter centriole (Fig. S5). Additionally, we stained cells for polyglutamylated α-tubulin (GT335), which marks the transition zone and proximal end of the axoneme (see Fig. S5A). GT335 staining was reduced in *Cp110^−/−^* compared with wild-type MEFs. With the transition zone marker Rpgr, we observed a partial overlap between ninein and Rpgr staining, and Rpgr was reduced in *Cp110^−/−^* MEFs compared with wild type. As predicted, immunostaining of wild-type and *Cp110^−/−^* MEFs with antibody against the IFT complex protein Ift88 did not detect any signal in mutant MEFs (Fig. 5D) because of the lack of cilia formation.

Talpid3 localizes to the basal body, associates with CP110 and is a positive regulator of cilia biogenesis. We therefore examined Talpid3 localization in mutant and control cells together with the ciliary rootlet marker rootletin (Yang et al., 2002). Talpid3 immunostaining in controls was directly adjacent to the rootlet, consistent with basal body staining. This localization pattern was unaltered in *Cp110^−/−^* neural tube and mesenchyme at E10.5 (Fig. S6), suggesting that Talpid3 is not dependent on CP110 for recruitment to, or association with, the basal body.

### Abnormal distribution of Rab11^+^ recycling endosomes in *Cp110^−/−^* MEFs

The distribution of recycling endosomes around the centrosomes and their interaction with the SDA component suggested a role for Rab11 in the docking of the ciliary vesicles to the distal end of the basal bodies (Hehnly et al., 2012). To test the integrity and distribution of the recycling endosomes in *Cp110^−/−^* MEFs, we examined the basal body marker γ-tubulin and the recycling endosome-localized protein Rab11 (Fig. 5E).

We noted an accumulation of Rab11-positive punctate staining around the majority of basal bodies (75%) in wild-type MEFs (Fig. 5E, left; Fig. S5D). However, Rab11 staining was reduced (to only 17% of basal bodies) in *Cp110^−/−^* MEFs (Fig. 5E, right), indicating aberrant distribution of recycling endosomes. A small fraction of *Cp110^−/−^* MEFs contained Rab11-positive vesicles; however, their distribution was random throughout the cell instead of centering around the basal bodies as in wild-type MEFs. This is consistent with Odf2 staining in *Cp110^−/−^* MEFs (Fig. 5B). Similarly, centrin 1 staining was reduced in *Cp110^−/−^* compared with wild-type MEFs (Fig. 5F).

### Aberrant ciliary vesicle docking and altered kinetics of MT array assembly in *Cp110^−/−^* MEFs

**Fig. 6.**
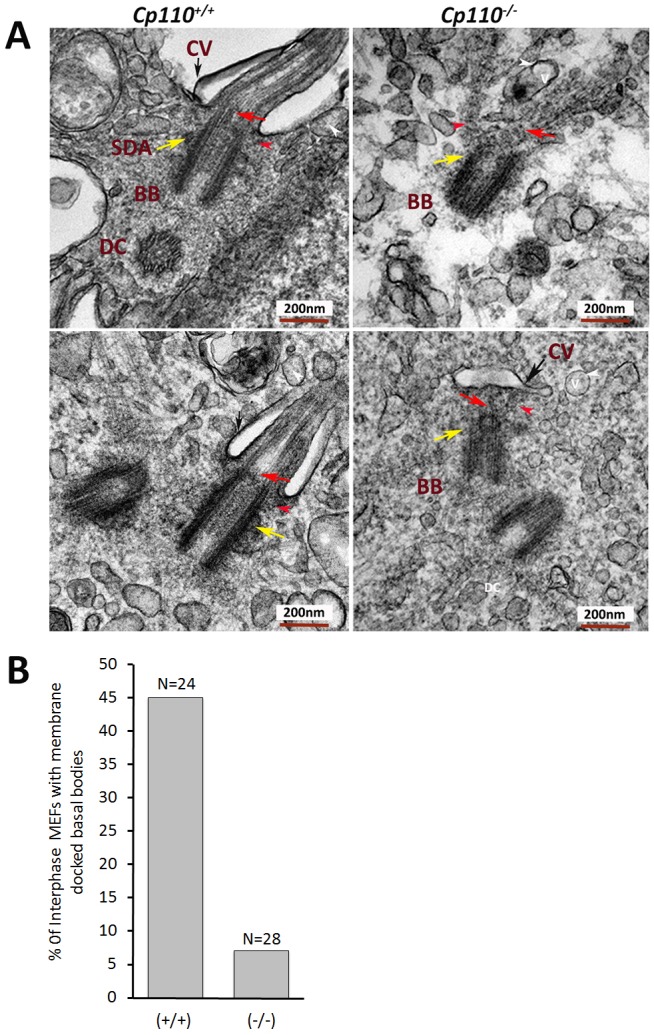
**TEM of basal bodies from wild-type and *Cp110^−/−^* MEFs.** (A) Wild-type MEFs show a highly organized mother centriole (MC) with well-defined dorsal appendage (DA, red arrowhead) and SDA (yellow arrow). SDAs anchor MTs, which help in trafficking vesicles (v). In wild-type MEFs, the transition zone (red arrow) was established and axonemal MTs emanated from the distal end of the MCs. A docked ciliary vesicle (CV, black arrow) is also detectable in wild-type MEFs. However, in *Cp110^−/−^* MEFs, SDAs are not visible (top right panel, yellow arrow) in the majority of basal bodies (BB) or are poorly developed (in less than 10% of MEFs) with a compromised transition zone (bottom right panel, red arrow). In rare instances, elongated axonemal MTs can be seen (top right panel) without ciliary vesicle docking. The accumulated cytoplasmic vesicles (white arrowheads) can be seen around MCs. We counted 24 longitudinal TEM images from wild-type and 28 TEM images from mutant MEFs and quantified the number of SDAs, the number of MEFs with basal bodies docked to the membrane, and the number of cells with transition zone formation through the vesicle. Unlike the wild type, a majority of basal bodies in *Cp110^−/−^* MEFs lacked DA and SDA, with no docked ciliary vesicle. In rare cases when observed (less than 7%, bottom right panel), the ciliary vesicle was docked far from the distal end. DC, daughter centriole. (B) The percentage of interphase basal bodies docked with membrane in wild-type and *Cp110^−/−^* MEFs, calculated from random TEM images.

To investigate the requirement of CP110 at the basal body (transition zone), we performed TEM to correlate reduced levels of SDA markers with structural components of the transition zone. In wild-type MEFs, SDAs are found associated with the MC. In most of the *Cp110^−/−^* MEFs, MC lacked SDAs and did not extend a ciliary axoneme. Furthermore, about half of all MCs in wild-type MEFs were docked to a ciliary vesicle and extended a ciliary axoneme (Fig. 6A). By contrast, less than 10% of *Cp110^−/−^* MEFs showed ciliary vesicle docking. Importantly, among the few mutant MCs that were docked to a vesicle, none had an extended transition zone into the ciliary vesicle. The quantified data are shown in Fig. 6B. We did not observe any significant difference in centrosome length in *Cp110* mutant MEFs compared with wild type (Fig. S5E). We also noted MTs emanating from the distal end of the MC in mutant cells (Fig. S7B). These data suggested that lack of CP110 might lead to the premature extension of axonemal MTs as a result of improper capping of the distal end of the MC, which in turn could contribute to the failure of ciliary vesicle docking to the MC and the absence of cilia in *Cp110^−/−^* MEFs.

To gain further insights into the MT assembly defect in *Cp110^−/−^* MEFs, we carried out MT regrowth assays to evaluate the nucleation and anchoring of cytoplasmic MT arrays (Fig. S8). Both wild-type and mutant MEFs started aster formation within 5 min of nocodazole removal; interestingly, MT nucleation appeared to have progressed faster in *Cp110^−/−^* MEFs. At 10 min, MTs can be seen in both wild-type and *Cp110^−/−^* MEFs. The increase in aster size is observed in both wild-type and mutant MEFs up to 15 min; however, MTs in *Cp110^−/−^* MEFs are no longer anchored at centrosomes after 30 min. MT arrays anchored to the centrosome can frequently be seen in wild-type MEFs after 30 min of regrowth. Our results suggest increased MT nucleation in the absence of CP110, possibly owing to an accumulation of pericentriolar materials as detected in other ciliary mutants. We also observed reduced anchoring of mature cytoplasmic MTs, which is likely to be related to structural defects in the SDA as noted earlier. These findings could provide one explanation for the reduced centering of Rab11-positive recycling endosomes around the centrosomes.

### Cell cycle defects in *Cp110^−/−^* MEFs

**Fig. 7.**
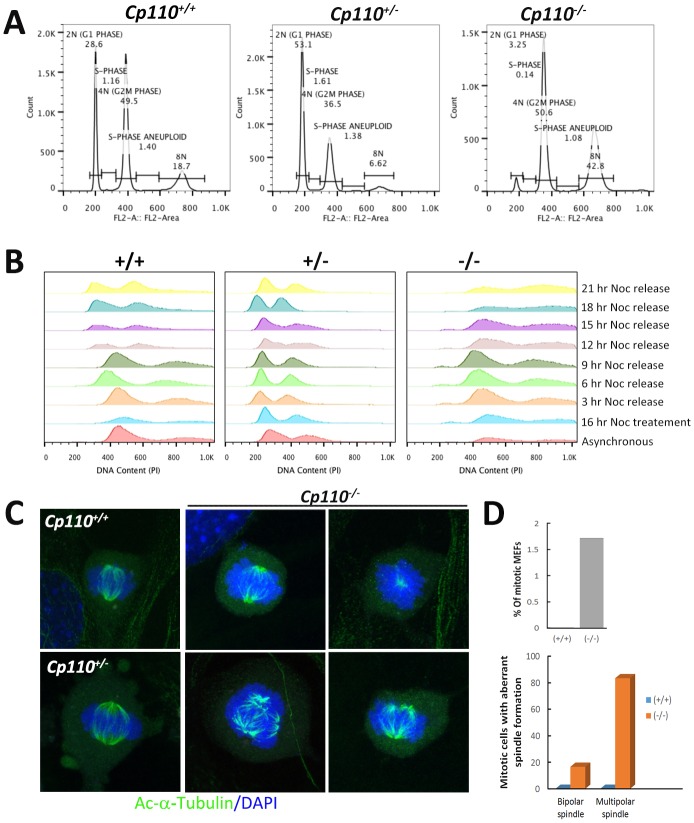
**Cell cycle analysis of wild-type and *Cp110^−/−^* MEFs.** (A) DNA content analysis of asynchronously growing wild-type and *Cp110^−/−^* MEFs was performed after fixing the cells in ethanol followed by staining with propidium iodide and FACS analysis. (B) Asynchronously growing wild-type, *Cp110^+/−^* and *Cp110^−/−^* MEFs were treated with nocodazole for 16 h and released for 3, 6, 9, 12, 15, 18 and 21 h, dissociated with trypsin, fixed in ethanol and stained with propidium iodide for DNA content analysis by FACS. (C) Analysis of the cell cycle defect in mutant MEFs. Mitotic cells from the wild-type, *Cp110^+/−^* and *Cp110^−/−^* MEFs were collected by mitotic shake off and plated on coverslips. Cells were grown for an additional 12 h before harvesting and fixation with PFA. Cells were stained for acetylated α-tubulin (green) to visualize the spindles. Nuclei were stained with DAPI. We noted acentrosomal, bipolar, as well as multipolar spindle orientation in *Cp110^−/−^* MEFs. (D) (Top) The percentage of mitotic cells among serum-starved wild-type (none) and *Cp110^−/−^* MEFs. (Bottom) The percentage of the mitotic cells with aberrant spindle formation. Three independent experiments were performed.

Impairment in centrosome biogenesis leads to defects at cell cycle checkpoints, which prevent cells with damaged DNA from replication (G1/S checkpoint) or entering mitosis (G2/M checkpoint) (Lambrus et al., 2015; Wong et al., 2015). The reported role of CP110 in centrosome duplication (Chen et al., 2002) led us to hypothesize a cell cycle progression defect during DNA replication and chromosome segregation at anaphase in *Cp110^−/−^* MEFs. To test whether the loss of CP110 affected the G1/S checkpoint, we performed an analysis of DNA content in asynchronized wild-type and *Cp110^−/−^* MEFs by fluorescence-activated cell sorting (FACS) after staining the nuclei with propidium iodide (Fig. 7A). We noted a drastic reduction in the percentage of G1/S phase cells in *Cp110^−/−^* MEFs compared with wild type. In addition, the mutant MEFs showed a ∼28% increase in cells at G2/M phase. Consistent with the original report on *Cp110* (Chen et al., 2002), our data suggested that deletion of CP110 results in abnormal cell division. We also observed an increase in aneuploidy (to ∼40%) in mutant MEFs, further confirming the defects in G2/M phase (Fig. 7A). In addition, there was an increase in G1 phase cells in *Cp110^+^^/−^* MEFs compared with the wild type at the expense of G2/M phase.

We then tested the growth and progression of the cells after arresting them with nocodazole in metaphase. We analyzed the DNA content in wild-type and *Cp110^−/−^* MEFs over a 24 h period after staining with propidium iodide (Fig. 7B). The wild-type MEFs recovered to normal cell cycle within 24 h of nocodazole release, whereas *Cp110^−/−^* MEFs were not able to return to normal cell cycle and instead showed accumulation of polyploid cells. Mutant cells appeared to die, probably owing to DNA damage, polyploidy and failure to segregate their centrioles.

To evaluate spindle formation, we stained asynchronously growing MEFs with anti-acetylated α-tubulin, which labels the spindle MTs (Fig. 7D). We rarely observed any dividing cells in serum-starved wild-type MEFs. However, ∼2% of the serum-starved *Cp110^−/−^* MEFs were still dividing (Fig. 7D, top). Further quantification of total mitotic nuclei in *Cp110^−/−^* MEFs revealed the presence of ∼17% bipolar and ∼80% multipolar spindle-containing cells (Fig. 7D, bottom), which was consistent with our *in vivo* data on ependymal epithelial cells (see Fig. S2A,B). Wild-type MEFs always showed bipolar orientation of the spindle at metaphase. By contrast, a number of cells with multipolar spindles were seen in *Cp110^−/−^* MEF culture. In addition, we often observed mutant cells with supernumerary, unseparated centrosomes (see Fig. S4B) in the center of the cell, leading to complete failure of mitotic spindle formation. MTs seemed to be originating from the center and moving towards the periphery (Fig. 7C, right). Most of the polyploid cells also had lagging chromosomes, a hallmark of chromosome breakage. Overall, the changes in cell cycle parameters and genome instability in the mutant cells are in accordance with the key role of CP110 in centrosome duplication and separation (Chen et al., 2002).

## DISCUSSION

Primary cilia develop from the mature MC (also known as the basal body) in cells at the G1/G0 phase of the cell cycle. This process is tightly controlled, in part through the actions of positive and negative regulators at the distal end of MCs, to ensure the correct timing of cilia formation. The centriole distal end capping protein CP110, along with its interacting partners Cep97 and Kif24, have been identified as negative regulators of cilia biogenesis (Kim and Dynlacht, 2013). Ciliogenesis is always accompanied by the removal of CP110 from the distal end of MCs, and ectopic expression of CP110 suppresses the formation of cilia. However, CP110 is suggested to have a more complex role in cilia biogenesis. For example, knockdown of CP110 may cause changes in basal body morphology but not lead to premature ciliogenesis. In extra-embryonic endoderm stem cells that normally lack cilia, CP110 caps both the mother and daughter centrioles and its knockdown results in elongated centrioles but not cilia formation (Bangs et al., 2015). Here, we demonstrate phenotypes reminiscent of human short rib-polydactyly syndrome, a form of ciliopathy with a strong skeletal defect (Huber and Cormier-Daire, 2012), in *Cp110^−/−^* mice. We also observed perturbation of Shh signaling in mutant embryos. Both findings are consistent with the loss of primary cilia or ciliary signaling and are validated by examination of cilia in mutant tissues and ciliogenesis in derived MEFs. TEM studies demonstrate that MCs in *Cp110^−/−^* cells remained at the cell center and lacked docked ciliary vesicles, indicating an early block in cilia formation. Thus, our study unexpectedly identifies CP110 as a positive regulator of cilia biogenesis.

Although the precise positive regulatory role of CP110 is unclear at this stage, we identified perturbations in the structure of SDAs that provide clues as to CP110 function. Cilia biogenesis requires recruitment of the recycling endosomes towards the basal bodies, where they interact with the SDA complex; this interaction is crucial for docking of recycling endosomes that carry the membrane and other components of the axoneme loaded in ciliary vesicles during cilia biogenesis (Graser et al., 2007; Ishikawa et al., 2005; Schmidt et al., 2012). Several proteins are reported to localize at SDAs (Chang et al., 2003; Gromley et al., 2003; Guarguaglini et al., 2005; Ishikawa et al., 2005; Mogensen et al., 2000), and loss of SDA core components causes perturbation in recycling endosome fusion to basal bodies (Hehnly et al., 2012). For example, the exocyst component Sec15 has been shown to interact with centriolin, and its depletion disrupts the organization or function of recycling endosomes at the MCs (Hehnly et al., 2012). Odf2, ninein, Cc2d2a, ε-tubulin and centriolin are core components of the SDA, and their loss leads to impaired cytoplasmic MT anchoring, disrupted recycling of endosome organization and function, and a reduced ability to form cilia (Chang et al., 2003; Dammermann and Merdes, 2002; Ishikawa et al., 2005; Veleri et al., 2014). We demonstrate in this study that cells lacking CP110 exhibit delocalization of ninein, Cc2d2a and Odf2 from basal bodies. Our results are consistent with previous findings that reduced Odf2 causes a decrease in the recruitment of Rab11-containing recycling endosomes to the SDA (Hehnly et al., 2012, 2013), leading to failure of cilia formation. Thus, CP110 appears to regulate the assembly of SDA core components, and its absence would lead to their mislocalization, thereby inhibiting the fusion of recycling endosomes to basal bodies.

An early step priming cilia biogenesis is MT nucleation and anchoring (Bornens, 2002). MTs dock to SDAs of the MC for anchoring the basal body. How the MTs are anchored to SDAs is poorly understood but is known to require ninein function. Trichoplein, a filament-binding structural protein, is also required for MT anchoring by binding to Odf2 and ninein (Ibi et al., 2011). Trichoplein and Odf2 levels dictate the localization of ninein at SDAs, suggesting the dependency of ninein on other proteins. Odf2 depletion reduces trichoplein levels, yet Odf2 levels are not dependent on trichoplein in the MC. Odf2 appears to be an integral component of the SDA core module, without which other SDA proteins fail to assemble. Reduced Odf2 and ninein staining in *Cp110^−/−^* MEF basal bodies will affect MT nucleation and anchoring, leading to abnormal docking of recycling endosomes and lack of cilia formation. Thus, our results suggest an integral role of CP110 in SDA core assembly during cilia biogenesis.

Another potential mechanism through which CP110 may positively control ciliogenesis might lie at the distal end of the MC. The centriole distal end capping protein complexes contain CP110 and are functionally multifaceted. Notably, the CP110-Cep97-Kif24 complex suppresses cilia formation, yet CP110 interacts with several positive regulators of ciliogenesis including Cep290 (Tsang et al., 2008) and Talpid3 (Kobayashi et al., 2014). Ablation of Talpid3 abolishes cilia formation, with basal bodies failing to dock to membranous vesicles (Bangs et al., 2011), a phenotype similar to that of the *Cp110^−/−^* mutant in our study. Our data showing that Talpid3 is not abolished or significantly altered in the mutant cells (it remains at the basal bodies) indicates that Talpid3 recruitment/association with the basal body does not depend on CP110.

In summary, while previous studies had reported its role in suppressing the formation of cilia from the basal body based on findings in cycling cells, our studies demonstrate a novel function of CP110 in promoting cilia biogenesis *in vivo*. Taken together, our studies highlight a dual and context-dependent role of CP110 during ciliogenesis and suggest that its function might be contingent upon available interacting proteins and the microenvironment within the cell. This testable hypothesis is open for future investigations.

## MATERIALS AND METHODS

### Antibodies

The following primary antibodies were used: rabbit anti-CP110 (gift from Eric Nigg (Biozentrum, University of Basel, Basel, Switzerland; 1:250), rabbit anti-CP110 (Proteintech, 2780-1-AP; 1:500), anti-acetylated α-tubulin (Sigma, T6793; 1:500), mouse anti-γ-tubulin (Sigma, T6557; 1:500), rabbit anti-γ-tubulin (Abcam, ab11317; 1:500), anti-GT335 (Adipogen, AG-20B-0020; 1:500), anti-Arl13b (Proteintech, 17711-1-AP; 1:500), anti-human ODF2 (cenexin; gift from Kyung Lee, National Cancer Institute, NIH, Bethesda, MD, USA; 1:50), anti-ninein L77 and L79 (gift from Michel Bornens, Institut Curie, Paris, France; 1:2500), rabbit and chicken anti-Rpgr (gift from Tiansen Li, National Eye Institute, NIH, Bethesda, MD, USA; 1:250), anti-Gli3 (gift from Tiansen Li; 1:300), goat anti-Gli3 (R&D Systems, AF3690; 1:1000), anti-Talpid3 (Proteintech, 24421-1-AP; 1:250), anti-Rab11 (Cell Signaling, #5589; 1:500), anti-pericentrin (Abcam, ab448; 1:500) and anti-β-actin (Abcam, ab8227; 1:1000). The secondary antibodies for immunofluorescence analysis were coupled to Alexa Fluor 488 or 568 (Molecular Probes).

### Generation of the *Cp110^−/−^* mouse

Mouse ESCs were transfected with a cassette containing *Cp110* exon 5 flanked by flox sites, followed by a neomycin selection marker flanked by FRT sites. Transfected ESCs were selected by neomycin, and positive clones confirmed for insertion of the cassette by PCR using a primer within the neomycin selection marker and another primer in the *Cp110* gene at either end. Cells were injected into developing embryos and implanted in pseudo-pregnant females. Chimeric mice obtained from the progeny were bred to wild-type C57BL/6 mice (Jackson Laboratory) and progeny from first generation validated by PCR. These mice were then bred with wild type for five generations to obtain *Cp110^flox/flox^* mice. The neomycin resistance cassette was removed by breeding with an actin promoter-driven Flp recombinase line (actin-Flp recombinase; Jackson Laboratory), and the progeny again bred with actin-Flp recombinase mice to ensure removal of the neomycin cassette. The *Cp110* heterozygous mice from this final flox line were bred with zona pellucida promoter-driven Cre mice (Zp3-Cre; Jackson Laboratory), which express Cre in developing oocytes. *Cp110 ^flox/+^* females that carried the *Zp3-Cre* gene were further bred with wild-type males to obtain *Cp110^+/−^* animals and, subsequently, *Cp110^−/−^* (null) mice. All experiments involving live animals conformed to the animal study protocol reviewed and approved by the National Eye Institute Animal Care and Use Committee.

### Skeletal preparations

Embryos were eviscerated and the skin removed, fixed overnight in 95% ethanol and stained in Alcian Blue solution (150 µg Alcian Blue, 800 ml 98% ethanol, 200 ml acetic acid) overnight. After several hours in 95% ethanol, the embryos were transferred to 2% KOH for 24 h and then stained overnight in Alizarin Red solution (50 µg Alizarin Red in 1 liter 2% KOH). The skeletons were cleared in 1% KOH/20% glycerol and stored in 50% ethanol/50% glycerol. A standard H&E staining method was used.

### Generation of MEFs

Embryonic fibroblasts were isolated from *Cp110^+/+^*, *Cp110^+/−^* and *Cp110^−/−^* embryos at E12.5, as described (Veleri et al., 2014). Briefly, embryos were finely minced by razor blade in a minimal amount of PBS and suspended in 1-2 ml trypsin-EDTA along with 100 Kunitz units DNase per ml/embryo and incubated with gentle shaking at 37°C for 15 min. The cell suspension was transferred to a 50 ml Falcon tube and two volumes of fresh DMEM were added. The suspension without any debris was centrifuged at 1000 rpm (180 ***g***) for 10 min. The cell pellet was suspended in warm DMEM containing serum and plated at one embryo equivalent per 10 cm dish. The cells were grown overnight, and the medium was changed the following day. MEFs are rapidly frozen for storage until use.

### Immunohistochemistry

For tissue sections, samples were prepared by fixing with 4% paraformaldehyde (PFA) at room temperature, followed by washing with 1× PBS and were kept overnight in 20% sucrose. Samples were embedded in OCT (Tissue-Tek) and 15-20 μm sections prepared. For MEF immunostaining, 80,000 MEFs were plated on a coverslip placed in each well of a 24-well plate with 500 µl of the medium. After overnight growth, cilia formation was induced with serum-free medium. The cells were grown to confluence for 48 h, washed with PBS, fixed with 4% PFA at room temperature for 15 min, and subjected to immunostaining with the antibodies described above. Confocal images were obtained using a Zeiss LSM 780.

### Immunoprecipitation and immunoblot analysis

Embryos were isolated from the timed mating of *Cp110^+/−^* parents. Females were sacrificed as described in the NIH animal protocol and E10.5 embryos were harvested and frozen at −80°C. After confirming the genotype, wild-type and *Cp110^−/−^* embryos were homogenized and cells were lysed in RIPA buffer (40 mM Tris-HCl pH 8.0, 150 mM NaCl, 2 mM EDTA, 1.0% DTT and 1% NP40) in the presence of protease inhibitor. Immunoprecipitation of Gli3 was performed by incubating 500 µl total embryo extract with 3 µl anti-Gli3 antibody overnight at 4°C followed by addition of 30 µl Protein A beads (Thermo Fisher Scientific, #10001D) to allow binding of the Gli3 protein complexes for 2 h. Protein A-bound Gli3 protein complexes were washed and then boiled in 60 µl RIPA buffer containing 1× SDS-PAGE sample buffer (Bio-Rad, 1610747). Proteins were resolved on 7.5% SDS-PAGE gel at 80 V and transferred to PVDF membranes at 25 V overnight at 4°C. Membranes were blocked with 5% non-fat milk and immunoblots were developed using anti-Gli3. β-actin was used as internal control in the case of total tissue extract.

### Quantification of signal intensity

Quantification of basal body-associated proteins by pixel intensity was performed as previously described (Veleri et al., 2014). Briefly, ImageJ (imagej.nih.gov/ij/) was used to quantify the pixel intensity of a defined square around the specific basal body-associated protein signal in wild-type and *Cp110^−/−^* MEFs. A similar area close to the protein signal was used to subtract background pixel intensity. Box plots were generated by uploading the .CSV files to BoxPlotR (http://boxplot.tyerslab.com/). The statistical analysis was performed using SPSS (IBM SPSS Statistics 20.0). The normality of data was tested with a normal quantile plot (Q-Q plot). Since most of our data were distributed asymmetrically, a Mann–Whitney *U*-test was applied for comparing wild-type and *Cp110^−/−^* samples. *P*≤0.05 was considered significant.

### Transmission electron microscopy

Serum-starved MEFs were fixed and processed for TEM as described (Gonda et al., 1976). Sections (80 nm) of cells in epoxy blocks were prepared with a Leica UCT ultramicrotome, mounted on 200-mesh copper grids and coated with carbon. The sections were imaged using a Hitachi H-7600 transmission electron microscope.
